# Rationale for Surrogate Endpoints and Conditional Marketing Authorization of New Therapies for Kidney Transplantation

**DOI:** 10.3389/ti.2022.10137

**Published:** 2022-05-20

**Authors:** Maarten Naesens, Alexandre Loupy, Luuk Hilbrands, Rainer Oberbauer, Maria Irene Bellini, Denis Glotz, Josep Grinyó, Uwe Heemann, Ina Jochmans, Liset Pengel, Marlies Reinders, Stefan Schneeberger, Klemens Budde

**Affiliations:** ^1^ Department of Microbiology, Immunology and Transplantation, KU Leuven, Leuven, Belgium; ^2^ Paris Translational Research Center for Organ Transplantation, Hôpital Necker, Paris, France; ^3^ Department of Nephrology, Radboud University Medical Center, Nijmegen, Netherlands; ^4^ Department of Nephrology and Dialysis, Medical University of Vienna, Vienna, Austria; ^5^ Department of Surgical Sciences, Sapienza University of Rome, Rome, Italy; ^6^ Paris Translational Research Center for Organ Transplantation, Hôpital Saint Louis, Paris, France; ^7^ University of Barcelona, Barcelona, Spain; ^8^ Department of Nephrology, Technical University of Munich, Munich, Germany; ^9^ Transplantation Research Group, Department of Microbiology, Immunology and Transplantation, KU Leuven, Leuven, Belgium; ^10^ Centre for Evidence in Transplantation, Nuffield Department of Surgical Sciences, University of Oxford, Oxford, United Kingdom; ^11^ Erasmus MC Transplant Institute, Department of Internal Medicine, University Medical Center Rotterdam, Rotterdam, Netherlands; ^12^ Department of General, Transplant and Thoracic Surgery, Medical University of Innsbruck, Innsbruck, Austria; ^13^ Department of Nephrology and Medical Intensive Care, Charité Universitätsmedizin Berlin, Berlin, Germany

**Keywords:** mortality, late graft failure, unmet medical need, morbidity, re-transplantation, clinical studies

## Abstract

Conditional marketing authorization (CMA) facilitates timely access to new drugs for illnesses with unmet clinical needs, such as late graft failure after kidney transplantation. Late graft failure remains a serious, burdensome, and life-threatening condition for recipients. This article has been developed from content prepared by members of a working group within the European Society for Organ Transplantation (ESOT) for a Broad Scientific Advice request, submitted by ESOT to the European Medicines Agency (EMA), and reviewed by the EMA in 2020. The article presents the rationale for using surrogate endpoints in clinical trials aiming at improving late graft failure rates, to enable novel kidney transplantation therapies to be considered for CMA and improve access to medicines. The paper also provides background data to illustrate the relationship between primary and surrogate endpoints. Developing surrogate endpoints and a CMA strategy could be particularly beneficial for studies where the use of primary endpoints would yield insufficient statistical power or insufficient indication of long-term benefit following transplantation.

## Introduction

The guideline CHMP/EWP/263148/06 of the European Medicines Agency (EMA) Committee for Medicinal Products for Human Use (CHMP), issued in 2008, identifies the primary composite endpoint for clinical trials in organ transplantation as recipient death, graft failure, biopsy-confirmed acute rejection, and graft (dys)function (1). Based on this composite endpoint, specific immunosuppressive drugs have received full (standard) marketing authorization for transplantation. However, CHMP/EWP/263148/06 does not mention any opportunities for other novel drugs to proceed to conditional marketing authorization (CMA), such as drugs that aim to improve long-term outcomes after kidney transplantation. This represents an area of considerable unmet medical need and restricts the development of novel treatments.

The present article proposes the rationale for surrogate endpoints for CMA, for novel kidney transplantation therapies; the paper also provides background data that illustrate the relationship between surrogate and primary endpoints, to support full marketing authorization.

CMA applications based on clinical trials using surrogate endpoints should not replace full marketing authorization applications based on studies using accepted primary endpoints. As discussed elsewhere in this Special Issue, graft rejection is acceptable as a primary endpoint for obtaining full marketing authorization by the EMA, because graft rejection is considered directly clinically meaningful, requiring therapies for rejection (2–4). Kidney function (incidence of end-stage renal disease, proportional decrease in eGFR, and annual decrease in eGFR—slope) is also well accepted by the EMA/CHMP as a primary endpoint to assess efficacy of medicinal products to slow progression of chronic renal insufficiency in chronic kidney disease. CHMP/EMA confirmed that this reasoning can be adopted for trials of kidney transplantation (5).

Rather, the CMA strategy and surrogate endpoints are suggested for studies where use of the accepted primary endpoints would yield insufficient statistical power or insufficient indication of long-term benefit. Applied to novel immunosuppressive agents, long-term benefit for kidney transplantation would equal decreased rates of late graft failure. It is therefore also important to have a very clear definition of late graft failure.

Here, we discuss the definition of late graft failure, and the rationale to consider late graft failure as a disease with unmet clinical need, allowing for CMA applications for novel therapies aimed at improving long-term kidney transplant outcomes. Endpoints that could be considered as surrogates for late graft failure are discussed separately in this Special Issue (6).

## Definition of Late Graft Failure

In discussions relating to the present article, we defined overall (all-cause) graft failure as a composite of two important primary endpoints: loss of graft function (i.e., return to dialysis or pre-emptive re-transplantation), and recipient death with a functioning graft.

We consider that using 1 year post transplantation as the border between early and late graft failure reflects current clinical research standards and epidemiological data. These illustrate a fundamental difference in general improvement of graft outcome within and beyond 1 year after transplantation (7).

In addition, a 1-year threshold for the definition of late graft failure could be appropriate, given that research standards usually consider primary endpoints at 6 months to 1 year following transplantation. This was the case for pivotal trials that supported the approval of immunosuppressive drugs (reviewed in (8)). The 1-year threshold for early versus late graft failure also reflects evidence that short-term graft outcomes (i.e, failure within the first year) improve over time (7); this was not the case for long-term graft failure, which was defined as any failure from 1 year post-transplant (7). In addition, in the Collaborative Transplant Study European data analyses (9), the 1-year graft survival rate improved considerably between 1986 and 1999, but no noteworthy improvement was seen for graft survival beyond the first year after transplantation. Lastly, there are relevant differences in the reasons for graft loss in different periods after transplantation; it is not the purpose of the present paper to discuss them (10).

## Rationale for CMA Applications for Late Graft Failure

The European Medicines Agency (EMA)-initiated concept of CMA (11) is an important tool for ensuring timely access to medicines in areas of unmet medical need. For CMA application, medicines for human use are eligible if they belong to at least one of the following three categories:

• Aimed at treating, preventing, or diagnosing seriously debilitating or life-threatening diseases
• Intended for use in emergency situations (less-comprehensive pharmaceutical and non-clinical data may also be accepted)
• Designated as orphan medicines, i.e., for the diagnosis, prevention, or treatment of a life-threatening or chronically debilitating condition that is rare (affecting <5 in 10,000 people in the European Union [EU]).

### Late Graft Failure: Seriously Debilitating, Life-Threatening

In kidney transplant patient populations, late graft failure is a common, seriously debilitating, and life-threatening condition; no specific measures are available for its prevention. Immunosuppressive drugs were primarily approved for prevention of early acute rejection, with limited impact on (late) graft failure (8). In Europe, death-censored graft failure rates (censoring for death with a functioning graft) beyond the first year post-transplantation have shown some improvement since the late 1980s (7, 9). However, ∼5% of grafts are still lost annually after the first year, including loss due to recipient death (7, 12, 13). On this basis alone, medicines that aim to prevent late kidney graft failure could be proposed for CMA.

Several aspects make late kidney graft failure a serious condition for which there is an unmet medical need. First, there is the requirement for dialysis reinitiation, which carries a heightened risk of mortality, comorbidities, and impaired health-related quality of life. Second, there is a high risk of human leukocyte antigen antibody (HLA) sensitization, which is associated with prolonged waiting time for repeat transplantation and further increased risk of dialysis complications. Third, increased risk of graft failure is observed after re-transplantation, which is related to heightened risk of antibody-mediated rejection (AMR) because of preformed antibodies against the first donor kidney (13, 14). In addition, increased morbidity and inferior outcomes after re-transplantation can result from diverse complications such as long waiting times, increased doses of immunosuppressive therapy, increased risk of infections and malignancies, high rates of acute rejection, and delayed graft function. Kidney graft failure is also associated with increasing the average waiting time for transplantation, due to relisting (15).

As of December 31, 2019, at the time ESOT was discussing this issue, ∼55,000 patients were on the transplantation waiting list in Europe (16), the vast majority of whom required kidney transplantation. Although ∼16% of transplantations performed in 21 European countries were re-transplants (9), data from Eurotransplant (which includes a different spread of countries) show that >20% of patients on the kidney waiting list required re-transplantation after failure of a prior graft (17). Longer waiting time on dialysis is an independent risk factor for death (18), and a considerable proportion of patients with graft failure die while waiting for re-transplantation. For example, in 2019, ∼10% of persons on the active Eurotransplant kidney waiting list were removed because they died or became unfit for transplantation (19).

While increasing longevity of kidney grafts could decrease the need for re-transplantation, importantly, the >20% of patients waitlisted for re-transplantation on Eurotransplant databases represents only those who are eligible for such procedures. Among European and US patients who experienced death-censored graft failure, 48% were waitlisted (median time 7.7 months) and 61% had HLA antibodies; most of the sensitized patients were not relisted for transplantation and remained on dialysis until death (20). A publication from Charité Hospital in Berlin found that between 1997 and 2017, 267 graft losses occurred in 254 patients, resulting in 117 (43.8%) relistings (21), of whom only 42 (35.9%) patients received a second transplant. At 5 years after graft loss, of the 254 patients, 49% had died, 27% were relisted, 14% were on dialysis and not relisted, and only 11% were re-transplanted (15).

Several studies demonstrate an increased mortality risk for patients who experience graft loss, compared with those with continued function (22–24) or those yet to receive a transplant (25). A study using competing-risk analysis confirmed a significantly increased all-cause mortality rate in patients relisted after graft failure compared with those awaiting a first transplant (16% vs. 11%; *p* = 0.033), with most deaths happening within 3 years of relisting (26). Prior transplant failure was associated with a 1.5-fold increased risk of mortality (95% confidence interval [CI] 1.01–2.2) (26).

However, a comparison of patients listed for first versus repeat transplantation does not account for the excess mortality rate seen in those who remain on lifelong dialysis after graft failure. Given that patients listed for re-transplantation are a selected population deemed capable of receiving another graft, it seems likely that those who are not relisted (primarily because of comorbidity and unacceptable risk) will have worse outcomes on dialysis. In addition, none of these analyses considers the burdens of returning to dialysis after failed transplantation, such as the costs associated with treatment (27), decreased ability to work and participate in society (28), and the psychological impact of returning to dialysis (29–31) (see also article by Tong et al. on patient reported outcome measures, in this Special Issue (32)).

### Late Graft Failure: An Orphan Indication?

In addition, late graft failure could be considered as an orphan indication, when its occurrence is calculated in absolute terms with the general population as reference. A hypothetical steady-state situation, where the same number of grafts are failing as are being transplanted, would result in ∼21,000 graft losses per 512 million inhabitants in the EU, equivalent to four graft losses per 100,000 people, per year. This may fulfill the definition of an orphan indication and would do so even if twice as many graft losses were to occur.

## Late Graft Failure: An Unmet Clinical Need

### Death With a Functioning Graft

Death of the recipient with a functioning graft is the most important reason for graft loss, and is usually a primary safety endpoint in studies of interventions that aim to prolong kidney transplant function. The main causes of death with a functioning graft are cardiovascular disease (CVD) and over-immunosuppression resulting in adverse events such as malignancy or infection (33–35). The fact that over-immunosuppression can cause death is obvious. Importantly, the relatively common side effects of immunosuppressants (e.g., diabetes mellitus, hypertension, altered lipid profile, and nephrotoxicity leading to low glomerular filtration rate) can also increase CVD risk (35). Graft function can also directly impact CVD risk and mortality, which provides further evidence for the pivotal role of good kidney function in both graft and patient survival (36, 37). The negative impact of poor kidney function on mortality (and CVD mortality in particular) is also seen in the general population (38, 39).

### Return to Dialysis/Re-Transplantation

**FIGURE 1:**
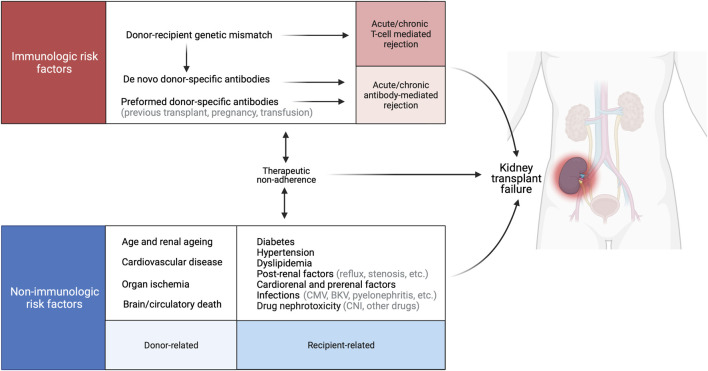
Causes of late allograft loss. Late graft failure is a multifactorial process that involves immunological factors related to the donor–recipient genetic mismatch, and nonimmunological factors that contribute to acute and chronic lesion development. BKV, BK virus; CMV, cytomegalovirus, CNI, calcineurin inhibitor. Created with BioRender.com.

Relative contributions of different pathological processes to graft failure have been evaluated (10, 33, 34, 40–42). Progression of fibrosis and accumulation of extracellular matrix i.e., interstitial fibrosis and tubular atrophy, IFTA) are key causes of graft loss. Fibrosis is thought to be mainly the consequence of nephron loss, and as aging is inevitably associated with a declining number of functioning nephrons, the quantity of nephrons might already be greatly reduced in grafts from marginal donors. After transplantation, nephrons can also be injured by immunological processes and/or other mechanisms (Figure 1) (43).

Increasing evidence suggests a continuous alloimmune response to the donor graft, despite modern immunosuppression, unrelated to the patient’s level of adherence to immunotherapy. The incidence of acute cellular (i.e., T cell-mediated [TCMR]) rejection in the early months after transplantation is ∼10% and rarely leads to immediate graft loss if treated appropriately, but TCMR is also an important, relevant, risk factor for long-term graft loss (10). Chronic TCMR has been described as a pathological entity and seems associated with impaired outcome, but its true prevalence and importance remain poorly defined (4, 44). By contrast, AMR diagnosis—and individual parameters of AMR—clearly show detrimental long-term effects on the graft (3, 10). B cells play key roles in AMR as antibody-producing cells and antigen-presenting cells for T cells with indirect allospecificity (12, 45). Poor adherence to medication is a major contributor to AMR development (10), highlighting that behavioral and social factors have important immunological consequences (43, 46). Poor adherence to complex medication regimens is common: it is estimated that up to 25% of patients have some degree of nonadherence, with severe nonadherence recognized as being a major contributor to late graft failure (10, 47). Poor adherence is associated with donor-specific antibody (DSA) development and poor control of metabolic factors (46).

As histologic studies show that progressive fibrosis is a major cause of late graft loss, and because calcineurin inhibitors (CNIs) are known to cause fibrosis, it was proposed that late graft loss might be partly attributable to CNI nephrotoxicity (10, 48), causing nephron injury and ultimately nephron loss with striped fibrosis. Studies have tested the hypothesis that minimizing the CNI dose, or avoiding these agents altogether, might improve long-term graft survival rates. Although some research suggested that avoiding CNIs did not cause safety issues and was associated with improvement in renal function over time, others indicated increased acute TCMR and DSA development in patients on CNI-sparing or CNI-free regimens and minimal, if any, improvement in renal function (49, 50). Thus, our understanding of the relative contribution of CNIs as the main cause of late kidney graft loss has evolved, and we recognize that competing risks (e.g., increased rate of rejection, or DSA development) might limit the success of CNI-sparing regimens.

After alloimmune-mediated injury, recurrence of native kidney disease in the transplanted organ is another common cause of graft loss (10, 51). Some native kidney diseases (e.g., focal segmental glomerulosclerosis or diseases associated with inherited complement defects) recur frequently, often early after transplantation and with poor ensuing graft survival. Although all kidney diseases are capable of recurrence, most do not strongly affect graft survival in the early years following transplantation. Of note, an elevated risk of late graft loss was observed in patients with recurring glomerulonephritis (12).

Nonimmunologic factors that contribute to post-transplantation nephron damage include brain death of the donor, poor donor management, and cold and warm ischemia times (52–54); delayed graft function (55); and infections (e.g., polyomavirus [BKV], cytomegalovirus, pyelonephritis) (10, 34). Kidney transplant recipients also usually have a high burden of comorbidities, some caused by chronic uremia before and during dialysis. Contributions of some modifiable CVD risk factors to the progression of native kidney disease have been demonstrated unequivocally, but their effect on graft survival remains unclear because interventional studies are scarce. In competing-risk analyses, smoking, systolic blood pressure, and hemoglobin concentration remain as independent predictors of graft failure or doubling of creatinine level (12). Standard immunosuppressive regimens increase the risk of diabetes and hyperlipidemia, which appear to accelerate graft rejection independently of the potential effects of lipids on the graft vasculature (12).

Other factors that contribute to graft failure are reflux nephropathy or obstruction due to ureteral stenosis (10). Finally, poor graft quality (e.g., graft having lower reserves because of older donor age or expanded criteria donors) with lower nephron mass transplanted is an important baseline risk factor for late graft failure, as described previously (10).

Clearly, late graft failure is often a multifactorial process: active/acute diseases are additive and coincide with cumulative chronic injury (10, 12, 34, 56, 57). This chronic injury can also have many causes, increasing the vulnerability of grafts to superimposed acute injury. Acute and chronic factors (as described above) can injure the nephron; once this basic functional unit of the kidney is irreversibly damaged, it cannot be replaced, and renal function deteriorates. Hyperfiltration and glomerular hypertension of the remaining nephrons can lead to a vicious circle, with further reduction in functioning nephrons, as seen in native kidney disease. Although late graft failure is a heterogeneous condition, the underlying disease processes often share a common clinical pathway of declining kidney graft function (indicated by a declining glomerular filtration rate) and/or increasing proteinuria, with a rise in chronic histological injury and fibrosis.

Several studies highlight the importance of progressive fibrosis as a key pathway to graft failure and a target for intervention, independent of the recognized role of late AMR in graft failure (42, 44). Biopsies late after transplantation are particularly dominated by nonspecific chronic lesions and IFTA without displaying concomitant inflammation (44). Beyond 5–10 years after transplantation, failures become increasingly biased toward IFTA, which therefore represents a key finding among identifying factors involved in late graft failure. It is precisely these late failures that have proven so resistant to advances in transplantation practice (7, 9). However, underlying causes of IFTA and progressive nephron loss remain poorly understood: the histopathologic picture is complicated by issues including rejection phenomena and chronic CNI nephrotoxicity, together with under-investigated but clearly detrimental factors such as aging, viral infections, reflux, and pyelonephritis.

Progressive IFTA in the absence of inflammatory disease is a process once known as “mysterious dysregulated fibrosis” (40, 58). New insights have illuminated this process, which can involve epigenetic mechanisms, resulting in constitutive fibroblast activation (59), drug nephrotoxicity (60, 61) and other pathophysiological aspects (e.g., oxidative stress or innate immune activation (62)). Therapies directed toward progressive IFTA, which are emerging in the management of native kidney disease, should have some value after transplantation (62).

### Unmet Needs: Interventions to Improve Late Graft Failure

Current immunosuppressive agents were approved for marketing based on studies with follow-up periods of <1 year. The approval of drugs that improved these short-term outcomes was based on research focusing on TCMR inhibition, which led to an important decline in early graft failure rates (7, 9) but did not substantially benefit long-term outcomes.

The impact of older immunosuppressive agents (e.g., cyclosporine) is not limited to short-term endpoints, however. Studies with ≥5-years follow-up periods, including cyclosporine withdrawal regimens, have demonstrated the effect of immunosuppressive drugs on long-term graft outcomes (63, 64). This suggests that different competing risks exist at different time points following transplantation. In addition, studies with tacrolimus have illustrated improved long-term outcomes compared with cyclosporine (65).

Very few randomized controlled trials (RCTs) have evaluated newer immunosuppressive agents (e.g., mTOR inhibitors, interleukin-2 receptor blockade, belatacept) with long-term graft survival as an endpoint. Extensions of the BENEFIT studies, reported at 7 and 10 years post transplantation (66, 67), demonstrated significantly lower risk of death or graft failure in the belatacept-treated group versus the cyclosporine-treated group, but only in standard criteria donor transplantations (67). Belatacept-treated patients had better outcomes despite having experienced more severe rejections (mainly TCMR) in the first year (66, 67), similar to findings of a study of early CNI withdrawal that included extensive follow-up (68). These studies clearly demonstrate the dissociation between TCMR and long-term outcome, suggesting that competing risks (e.g., cyclosporine toxicity, differences in metabolic profile, *de novo* DSA development) are more important than TCMR for long-term transplantation success.

Other studies had extended follow-up (beyond 1 year) after transplantation, comparing regimens of immunosuppressive agents that were approved based on short-term data. Although graft function sometimes improved over time, this did not reduce the rates of long-term graft failure (68, 69). Sometimes, worsening graft function and long-term graft survival rates were observed for the innovative regimen (70), which supports the hypothesis that long-term graft survival is affected by different competing risks at different time points. The complex reasons for graft loss (10), and the paucity of RCTs investigating the translation of short-term results into long-term survival benefits, highlight the difficulties in powering such trials sufficiently. Interpretation of long-term follow-up data is also confounded by frequent conversions to new, different immunosuppressive regimens.

## Surrogate Endpoints for CMA Applications for Late Graft Failure

If CMA applications for novel drugs aiming at preventing or treating late graft failure are admissible to the EMA, the next discussion relates to the choice of the endpoints to be used for the required clinical trials. Graft failure is a highly relevant hard endpoint in clinical studies, but it is a late endpoint. This hampers the feasibility of using graft failure as an endpoint in clinical trials that aim at improving late graft failure rates.

Surrogates for late graft failure are therefore needed but require robust definitions. A good surrogate endpoint should fulfil four criteria: 1) The disease process is sufficiently understood; 2) The surrogate endpoint has biologic plausibility; 3) The strength of the consistency supports the relationship between the surrogate marker and outcome; 4) Treatment effects on the surrogate endpoint predict treatment effects on the clinical outcome of interest.

Kidney graft function and combined functional markers, donor-specific HLA antibodies and composite scores could be considered as surrogate endpoints, but do not fulfill all these criteria. For a detailed discussion on the potential acceptability of these surrogate endpoints for late graft failure, we refer to another manuscript in this Special Issue (6).

## From Conditional to Full Marketing Authorization

After successful application for CMA of a product aimed at improving long-term graft survival, conversion to full marketing authorization is necessary, based on a post-marketing confirmatory commitment.

ESOT sees different options for this conversion of CMA to full marketing authorization. For example, applicants could consider requests for full marketing authorization based on long-term registration studies with accepted primary endpoints relating to graft rejection (2–4) function (5) and/or graft failure. Applicants could also consider requesting full marketing authorization based on comprehensive high-quality evidence from open-label study data, comparing findings to appropriate historic controls.

Alternatively, applicants could base the comprehensive evidence for full marketing authorization requests on good-quality data from registration studies, utilizing real-world data. Indeed, the EMA has already considered data from two other registries suitable for their decision-making processes: the European Cystic Fibrosis Society patient registry and the Cellular Therapy module of the European Blood and Marrow Transplant registry. The EMA Patient Registries Initiative (71) offers guidance on this topic. Of note, ESOT emphasizes that currently no European registries in kidney transplantation could be used as basis for requesting full marketing authorization.

A final option could be to use data from a qualified surrogate endpoint as a source of comprehensive evidence for a full marketing authorization request. Although CHMP/EMA has suggested to initiate a formal Qualification of Novel Methodologies procedure for e.g. the finalized iBox model (69) as a surrogate marker, this qualification is not yet achieved. The status and path toward formal qualification of composite scores as potential surrogate endpoints is discussed separately in this Special Issue (6).

Each of the above options for post-marketing commitments seems unsatisfactory at present, in the field of kidney transplantation. This may hamper the current admissibility of CMA applications for therapies aiming at reducing the incidence and burden of late kidney transplant failure. The results of formal qualification procedures are eagerly awaited and will hopefully change the landscape in future.

## Conclusion

• Late graft failure (loss of graft function >1 year post transplant) is a condition with unmet medical need. Therefore, CMA should be considered for interventions that demonstrate potential benefits:

 ○ Late graft failure is a seriously debilitating, life-threatening disease for which no specific preventive or treatment options are available.
 ○ CMA of therapies aimed at preventing late graft failure could be based on trials that show benefit on a validated surrogate endpoint for graft failure.

• For drugs aimed at reducing late graft failure, applying for CMA could be considered.

 ○ CMA procedures facilitate timely access to new therapies.
 ○ Confirmatory post-marketing commitments will be needed to convert CMA to full marketing authorization.

### Scientific Advice From the Committee for Medicinal Products for Human Use (CHMP) of the European Medicines Agency (EMA) Regarding These Conclusions

• The CHMP agreed that improving long-term outcome after kidney transplantation is an area of unmet medical need; arguments for orphan designation of late graft failure were not followed.
• Should a novel therapy be proposed for CMA, the product will need to fulfil all of the following four criteria at the time CMA is considered: 1) positive benefit/risk balance; 2) it is likely that the applicant will be able to provide comprehensive data later; 3) unmet medical need is fulfilled; and 4) the benefit to public health of the medicinal product’s immediate availability on the market outweighs the risk due to need for further data.
• Criteria for CMA will be reviewed for specific data submitted; CMA cannot be granted *a priori* for any given product or indication.
